# Long-term prognosis of lupus nephritis: comparison between pediatric, adult, and advanced age onset

**DOI:** 10.3389/fimmu.2025.1531675

**Published:** 2025-03-13

**Authors:** Marta Calatroni, Simeone Andrulli, Federico Doti, Federica Bello, Giovanni De Vivo, Antonio Mastrangelo, Nicoletta Del Papa, Tommaso Schioppo, Laura Locatelli, Francesco Reggiani, Gabriella Moroni

**Affiliations:** ^1^ Department of Biomedical Sciences, Humanitas University, Milan, Italy; ^2^ Nephrology and Dialysis Division, IRCCS Humanitas Research Hospital, Milan, Italy; ^3^ Associazione Italiana Ricercare per Curare ODV ETS (AIRpC), Lecco, Italy; ^4^ Department of Medicine and Surgery, University of Milano-Bicocca, Monza, Italy; ^5^ Department of Experimental and Clinical Medicine, University of Firenze, Florence, Italy; ^6^ Internal Interdisciplinary Medicine Unit, Careggi University Hospital, Florence, Italy; ^7^ Humanitas University, Milan, Italy; ^8^ Department of Pediatric Nephrology, Dialysis and Transplant Unit, Fondazione Istituti di Ricovero e Cura a Carattere Scientifico (IRCCS) Ca’ Granda, Maggiore Policlinico Hospital, Milan, Italy; ^9^ Scleroderma Clinic, Unità Operativa Complessa (UOC) Clinica Reumatologica, ASST Pini-Centri Traumatologici Ortopedici (CTO), Milano, and Department of Clinical Sciences and Community Health, Università degli Studi di Milano, Milan, Italy; ^10^ Medicina Generale II, Ospedale San Paolo, ASST Santi Paolo Carlo, Milan, Italy

**Keywords:** lupus nephritis, childhood lupus nephritis, acute kidney disease, chronic kidney disease, older age lupus nephritis

## Abstract

**Background and hypothesis:**

Lupus nephritis (LN) presents with varied outcomes depending on the age at diagnosis. We aimed to evaluate long-term kidney survival across three age groups.

**Methods:**

Patients were categorized based on their age at lupus nephritis diagnosis: ≤18 years (childhood), >18 to <45 (adulthood), and ≥45 years (elderly). The three groups’ CKD (eGFR <60 ml/min/1.73 m^2^ for at least 3 months) or death-free survival was estimated using Kaplan–Meier curves and compared with the log-rank test. To evaluate the independent prognostic role of age, adjusted for other predictors of chronic kidney disease (CKD) or death, we used multivariate Cox regression analysis.

**Results:**

This retrospective cohort study analyzed 260 patients followed for a median of 14.8 years. Of them, 46 (17.7%) were <18, 173 (66.5%) >18 and <45, and 41 (15.8%) ≥45 years old. 46% of elderly vs. 32.6% of children and 24.3% of adults had acute kidney disease (AKD) at diagnosis (P=0.02). Children had more active SLE, whereas the elderly had more chronic damage and hypertension. At 5, 10, and 20 years, CKD or death-free survival rates were 95.3%, 92.5%, and 88.4% in children; 98.2%, 90.1%, and 82.6% in adults; and 87.5%, 67.8%, and 53.5% in the elderly, respectively. Survival in elderly patients was significantly worse compared with children and adults (P= 0.001), whereas survival rates between children and adults were comparable (P = NS). At multivariate analysis, when the chronicity index was excluded from the model, older age emerged as an independent predictor of CKD or death (relative risk, RR: 3.278; CI: 1.402–7.662; P=0.006), with AKD (RR: 2.930; CI: 1.674–5.130; P<0.001), arterial hypertension (RR: 3.692; CI: 1.844–7.389; P<0.001), SLICC >0 (RR: 1.824; CI: 1.155–2.881; P=0.01), and failure to achieve complete remission at 1 year (RR: 4.784; CI: 2.355-9.716; P<0.001).

**Conclusion:**

While children and adults demonstrate comparable long-term kidney survival, elderly patients face significantly worse outcomes due to advanced chronicity and systemic damage. These findings highlight the need for tailored interventions in late-onset LN. Older-onset LN, in fact, was an independent predictor of CKD or death together with AKD, arterial hypertension, SLICC >0, and no remission at 1 year.

## 1 Introduction

Systemic lupus erythematosus (SLE) mainly affects young women, although 15%–20% of cases are diagnosed before 18 years (1, 2) and up to 20% in the elderly (3, 4).

Based on available data, childhood-onset SLE is often more aggressive, with higher rates of renal, neuropsychiatric, and hematological issues compared with adult-onset SLE (5, 6). In contrast, older-onset SLE is usually milder, with fewer kidney involvement but with more comorbidities (3, 7, 8).

Lupus nephritis (LN) is a severe complication, and age influences the clinical expression and disease severity (5, 9). Compared with childhood patients, late-onset patients seem to have an insidious onset, fewer active lesions at kidney biopsy, but a higher chronicity score (10). Several studies have shown that children had more severe renal outcomes than late-onset patients, with more progression to dialysis (11). Other studies suggest that pediatric patients may have good long-term renal prognosis nowadays, despite severe renal presentation (10).

Our study aims to evaluate in three age groups (childhood, adults, and late-onset LN) a) the differences in presentation and long-term kidney and patient survival and b) factors associated with the development of chronic kidney disease (CKD) and CKD or death in the entire cohort.

## 2 Materials and methods

### 2.1 Study cohort

This is a retrospective observational study that included LN patients diagnosed from 1980, followed until December 2023 at a single nephrological center and prospectively followed. Inclusion criteria were i) classification of SLE according to the American College of Rheumatology (ACR) criteria (12, 13); ii) demographic, clinical, and immunological assessments at LN diagnosis, 1 year after the start of therapy and last observation; iii) a minimum follow-up of 12 months post-therapy initiation; iv) any of the kidney syndromes defined below. Patients were categorized by age at clinical LN diagnosis into three groups (≤18 years: childhood onset, >18 and <45 years: adulthood onset, and ≥45 years: late onset that corresponds to the average menopausal age of our cohort).

The exclusion criterion is chronic kidney disease (CKD) at diagnosis of LN.

### 2.2 Ethical approval

Approved by the Ethics Committee of IRCCS Humanitas Rozzano, Milan, Italy (protocol NEF0032023), and by Declaration of Helsinki. All patients provided informed consent for the scientific use of their anonymized data. Patient or public involvement in the research was not applicable.

### 2.3 Data collection and definitions

An electronic database has been in use since 1990 in which data of patients diagnosed from 1980 to 1990 were implemented and then collected in the database at each clinical visit. At diagnosis, we collected data regarding organ SLE involvement and the laboratory findings reported in **Table 1A**. SLE disease activity was assessed using the Systemic Lupus Erythematous Disease Activity Index 2000 (SLEDAI-2K) (14, 15). Damage was assessed by systemic lupus international collaborating clinics American College of Rheumatology Damage Index (SDI) (16).

**Table 1A:** Demographic, clinical, histological, and therapeutic characteristics at baseline, in those who developed lupus nephritis at <18 years, between >18 and <45 years, and >45 years of age.

	All patients	Missing data	LNdiagnosis ≤18 years	LNdiagnosis >18 and <45 years	LNdiagnosis ≥45 years	P among the three age groups*	P between young and old class*
Demographics and clinical renal features at lupus nephritis diagnosis							
	260	260	46 (17.7%)	173 (66.5%)	41 (15.8%)		
Females, n (%)	229 (88.1%)	0	37 (80.4%)	156 (90%)	36 (87.8%)	0.19	0.35
Caucasians, n (%)	257 (99%)	0	43 (98%)	173 (100%)	41 (100%)	<0.01*	0.35
Age at SLE diagnosis (years)	24 (18, 35)	0	15.0 (12, 17)	25.0 (20, 31)	48.0 (44, 54)	<0.01*	<0.01*
Age at LN diagnosis (years)	28 (20.8, 39.2)	0	15 (13, 17)	28.0 (24, 36)	51.0 (47, 60)	<0.01*	<0.01*
Time between SLE and LN diagnosis (months)	6 (0, 58.5)	0	0 (0, 4.75)	9 (0, 71)	19 (0, 132)	<0.01*	<0.01*
Diagnosis of SLE and LN simultaneous or within 1 year n (%)	152 (58.5%)	0	39 (74.78%)	94 (54.33%)	19(43.34%)	<0.01*	<0.01*
Serum creatinine (mg/dl)	0.9 (0.7, 1.3)	0	0.82 (0.7, 1.2)	0.90 (0.7, 1.23)	1.00 (0.78, 1.6)	0.98	0.02*
eGFR (ml/min/1.73 m^2^; according to Schwartz for pts <18 years, according to CKD-EPI for >18 years)	87.4 (55.4, 112.4)	0	77.68 (53.3, 95)	90.97 (61.3, 118.3)	68.2 (37.5, 91.4)	<0.01*	0.09
Acute kidney damage** of them with proteinuria >3.5 g/day	76 (29.2%) 45	0	15 (32.6%) 12	42 (24.3%) 26	19 (46.4%) 8	0.02*	0.19
Proteinuria, g/day	3.5 (2.00, 5.5)	1 (0.4%)	3.60 (1.85, 5.06)	3.55 (2.00, 5.48)	3.2 (1.79, 4.92)	0.26	0.54
Nephrotic syndrome, n (%)	83 (31.9%)	1 (0.4%)	12 (26.1%)	60 (34.7%)	11 (26.8%)	0.43	0.93
Isolated urinary abnormalities	101 (38.9%)	0	19 (41.3%)	71 (41.0%)	11 (26.8%)	0.22	0.15
Red blood cells at urinary sediment analysis (n°/HPF 400rpm)	13 (5, 40)	1 (0.4%)	15 (5, 45)	12 (4.5, 37.5)	15 (3.5, 30)	0.04*	0.39
Arterial hypertension, n (%)	131 (50.4%)	1 (0.4%)	19 (41.3%)	80 (46.2%)	32 (78%)	<0.01*	<0.01*
Immunological and clinical SLE manifestations at LN diagnosis							
C3, mg/dl	58.0 (46, 78)	12 (4.6%)	55 (34.75, 80)	56 (46, 73)	68 (50, 83)	<0.01*	0.08
C4, mg/dl	10 (6, 14)	13 (5%)	8 (4, 13.5)	10 (6, 14)	10 (5, 18)	<0.01*	0.08
Antiphospholipid antibodies positivity, n (%)	61 (23.5%)	20 (7.8%)	15 (32.6%)	37 (21.4%)	9 (22%)	0.27	0.27
Anti SSA positivity, n (%)	83 (31.9%)	40 (15.4%)	7 (15.2%)	55 (31.8%)	21 (51.2%)	<0.01*	<0.01*
Anti SM positivity, n (%)	47 (18.1%)	39 (15%)	10 (21.7%)	29 (16.8%)	8 (19.5%)	0.71	0.80
Anti RNP positivity, n (%)	54 (20.8%)	40 (15.4%)	8 (17.4%)	37 (21.4%)	9 (22%)	0.82	0.59
Fever, n (%)	130 (50.0%)	5 (1.9%)	32 (69.6%)	86 (49.7%)	12 (29.3%)	<0.01*	<0.01*
Arthralgia, n (%)	200 (76.9%)	4 (1.5%)	35 (76.1%)	131 (72.7%)	34 (82.9%)	0.61	0.43
Lymphadenopathy, n (%)	43 (16.5%)	6 (2.3%)	6 (13%)	29 (16.8%)	8 (19.5%)	0.71	0.41
Skin manifestations, n (%)	159 (61.2%)	4 (1.5%)	30 (65.2%)	108 (62.4%)	21 (51.2%)	0.34	0.19
Serositis, n (%)	67 (25.8%)	4 (1.5%)	10 (21.7%)	48 (27.7%)	9 (22%)	0.59	0.98
SLEDAI	15 (10.75, 18)	4 (1.5%)	14 (9,19)	15 (11, 18)	14 (12, 18)	0.02*	0.79
SDI	0 (0, 1)	4 (1.5%)	0 (0, 0.25)	0 (0, 1)	0 (0, 1)	1	0.01*
Histopathological classes, activity, and chronicity index at kidney biopsy							
Class I/II/V/V+II, n (%)	58 (23.5%)	13 (5%)	11 (23.9%)	39 (24.1%)	8 (20.5%)	0.89	0.70
Class III/IV and mixed, n (%)	188 76.1%)	13 (5%)	35 (76.1%)	122 (75.3%)	31 (79.5%)	0.85	0.70
Class VI, n (%)	1 (0.4%)	13 (5%)	0	1 (0.6%)	0	/	1
Activity index	6 (3, 9)	60 (23%)	6 (2, 8)	5 (3, 9.75)	6 (4, 9)	0.02*	0.56
Chronicity index	1 (0, 3)	60 (23%)	1 (0, 2)	1 (0, 3)	3 (1, 4)	0.5	<0.01*
Immunosuppressive therapy induction							
Methylprednisolone pulses, n (%)	215 (82.7%)	0	38 (82.6%)	139 (80.3%)	38 (92.7%)	0.17	0.15
Immunosuppressive therapy, n (%)	219 (84.2%)	1 (0.4%)	39 (84.8%)	142 (82.1%)	38 (92.7%)	0.24	0.25
Cyclophosphamide, n (%)	116 (44.6%)	1 (0.4%)	22 (47.8%)	77 (44.5%)	17 (41.4%)	0.83	0.55
Azathioprine, n (%)	26 (10%)	1 (0.4%)	7 (15.2%)	15 (8.7%)	4 (9.8%)	0.42	0.44
Mycophenolate mofetil, n (%)	53 (20.4%)	1 (0.4%)	6 (13.0%)	34 (19.7%)	13 (31.7%)	0.90	0.04*
Cyclosporine, n (%)	12 (4.6%)	1 (0.4%)	3 (6.5%)	8 (4.6%)	1 (2.4%)	0.66	0.36
Rituximab, n (%)	11 (4.2%)	1 (0.4%)	1 (2.2%)	7 (4%)	3 (7.3%)	0.48	0.25
Immunosuppressive maintenance therapy							
Immunosuppressive therapy, n (%)	167 (64.2%)	1 (0.4%)	28 (60.9%)	109 (63%)	30 (73.2%)	0.41	0.22
Azathioprine, n (%)	53 (20.4%)	1 (0.4%)	10 (21.7%)	35 (20.2%)	8 (19.5%)	0.96	0.80
Mycophenolate mofetil, n (%)	95 (36.5%)	1 (0.4%)	14 (30.4%)	60 (34.7%)	21 (51.2%)	0.09	0.05*
Cyclosporine, n (%)	18 (6.9%)	1 (0.4%)	4 (8.7%)	13 (7.5%)	1 (2.4%)	0.45	0.21

n°, number; pts, patients; SLE, systemic lupus erythematosus; LN, lupus nephritis; eGFR, estimated glomerular filtration rate; SLEDAI, systemic lupus erythematosus disease activity index; SDI, SLICC Damage Index; SLICC, systemic lupus international collaborating clinics American College of Rheumatology Damage index. If not differently specified data are expressed as median (first and third quartile).

*P refers to T-test for continuous measurements between the first and third age class, Kruskal–Wallis H test for continuous measurements among the three classes, and to Chi-squared test for discrete measurements, with P<0.05.

**Acute kidney damage: eGFR <60 ml/min/1.73/m^2^ for <3 months, hematuria (urinary red blood cells >20/high-power field [HPF]), and/or erythrocyte casts, proteinuria ≥0.5 g/day (Levey AS, Eckardt KU, Dorman NM, et al. Nomenclature for kidney function and disease: report of a Kidney Disease: Improving Global Outcomes (KDIGO) Consensus Conference. Kidney Int 2020; 97:1117-29).

Kidney syndromes were defined as follows: a) *acute kidney disease (AKD)*: eGFR <60 ml/min/1.73/m^2^ for <3 months, hematuria (urinary red blood cells >5/high-power field [HPF]) and/or erythrocyte casts, proteinuria ≥0.5 g/day (17); b) *nephrotic syndrome:* proteinuria ≥3.5 g/day, serum albumin ≤3 g/dl, and eGFR ≥ 60 ml/min; c) *isolated urinary abnormalities:* proteinuria <3.5 and ≥0.5 g/day and/or microscopic hematuria (urinary red blood cells >5/high-power field HPF) and eGFR ≥60 ml/min“ (18); d) *complete renal remission:* stable eGFR if normal at diagnosis or at least 10% improvement if AKD ad diagnosis, proteinuria <0.5 g/day, and inactive urinary sediment; e) *partial renal remission:* eGFR as for complete remission, and reduction in proteinuria <3.5 g/day with a reduction of at least 50% from peak value (19); f) *no response:* all the other situations; g) *chronic kidney disease (CKD)*, eGFR <60 ml/min per 1.73 m^2^ (23) for at least 3 months (17); h) *arterial hypertension*: the mean of three consecutive measurements of systolic blood pressure >140 mm/Hg and/or diastolic blood pressure >90 mm/Hg in a sitting position.

*eGFR* was calculated with the CKD EPI formula for patients >18 years old (20) and the modified Schwartz formula for pediatric patients (≤18 years) (21). Proteinuria measured by benzethonium chloride in the urine collected over 24 h expressed as g/24 h.

Kidney biopsies were classified according to the International Society of Nephrology/Renal Pathology Society (ISN/RPS) criteria (22). The activity and chronicity indices were based on the last revision of the SLE classification (23).

*The start of the study* is the date of clinical diagnosis of LN.

*The first objective* of the study was as follows: differences in clinical/histological presentation, CKD, and CKD or death development among the three subgroups of LN patients—children, adults, and elderly. We decided to use CKD as the primary endpoint of interest because the low number of patients who achieved end-stage kidney disease (ESKD, eGFR <15 ml/min) reduced the power of identification of potential predictors of kidney survival.

To assess the role of age groups in LN prognosis, we added a *second objective*: the evaluation, in the whole cohort, of the predictors of CKD development, and CKD or death. The possible confounding role of covariates and factors like baseline kidney function, age, gender, proteinuria before biopsy, proteinuria reduction during the follow-up, and number and type of immunosuppressive drugs were evaluated.

### 2.4 Statistical analysis

Demographic and clinical data were expressed as absolute numbers and percentages, for categoric variables, and continuous variables were reported as mean and standard deviation (SD) or median and interquartile range (IQR) depending on their distribution. A comparison of continuous variables between groups was conducted using the T-test for continuous variables with normal distribution. Non-parametric Mann–Whitney U test or the Kruskal–Wallis H test for two or more independent samples, respectively, for continuous variables without normal distribution. The chi-squared test was employed to compare categorical or dichotomized variables among groups of patients. Kaplan–Meier survival curves for each outcome were grouped by age category. Survival free from CKD was calculated and survival differences among the three groups were assessed with the log-rank test. For inferential purposes, multivariate survival Cox regression analysis was performed, using the listed above two outcome variables (**Supplementary Table 1**). Covariate parametrization and contrasts were defined according to the categorical, discrete, or continuous nature of predictor, i.e., for each categorical variable coding, an indicator dummy variable is used (1 if present and 0 if absent). To investigate the role of the various covariates, a step-by-step iterative approach was used starting from the full model. For each selected covariate, we reported beta coefficients, exponential beta coefficients (as an estimate of relative risk), and their 95% confidence intervals. Covariates were selected for the multivariate Cox regression model using a backward stepwise approach, based on the likelihood ratio test with entry and removal criteria set at P = 0.1 and P = 0.05, respectively. In the multivariate analyses, the missing data excluded the corresponding records from the analysis. All the analyses were performed using the Statistical Package for Social Sciences (SPSS for Windows, version 23.0).

## 3 Results

### 3.1 Clinical/histological characteristics at diagnosis and outcome

A total of 260 patients were included in this study, of which 99% were Caucasian and 229 (88.1%) were women. Baseline demographics and clinical features are reported in **Table 1A**. At lupus nephritis onset, 76 patients (29.2%) presented with acute kidney disease (AKD) (of them, 45 also had proteinuria >3.5 g/day), 85 patients (32.7%) had nephrotic syndrome with normal renal function, and the last 99 patients (38.1) had isolated urinary abnormalities. 50% of patients had arterial hypertension. A total of 247 out of 260 patients (95%) received a kidney biopsy in a median of 2.01 months (0.31–7.76) after the clinical diagnosis of LN. In the remaining 13 patients, the diagnosis of LN was done on clinical grounds with AKD in three patients, nephrotic syndrome in five patients, and isolated urinary abnormalities in the remaining five patients. In these 13 patients, kidney biopsy was not performed due to the patients’ refusal to undergo a kidney biopsy or because of clinical contraindications. Kidney biopsies revealed the following distribution of histological classes: class III in 23.9% (n = 59, including 28 patients with concomitant class V lesions), class IV in 52.2% (n = 129, including 22 with concomitant class V), and class V in 19.4% (n = 48). A smaller proportion had class II (3.6%, n=9), class I (0.4%, n=1), and class VI (0.4%, n= 1). These findings highlight the predominance of advanced nephritis (classes III and IV) in this cohort.

The median activity and chronicity index were 6 (3–9) and 1 (0–3), respectively. At diagnosis, 186 patients (71.5%) had no chronic damage (SDI=0), whereas the median SLEDAI was 15 (10.78-18).Initial treatment, administered at diagnosis of LN based on clinical SLE extrarenal and renal manifestations as well as on the results of kidney biopsy, consisted of three intravenous methylprednisolone pulses (iv MPP) in 82.7% of patients and in 1 mg/kg/day of oral prednisone in the other patients. Oral prednisone was progressively tapered to a maintenance of 7.5–5 mg/day. Immunosuppressive drugs were added in 84.2% of patients. Rituximab was given to 4.2% of patients. hydroxychloroquine was taken by 66 patients (25.4%) at baseline.

One year after treatment initiation, complete renal remission was achieved by 54.6% of patients, partial renal remission by 25.8%, and 14.2% were non-responders (**Table 1B**).

**Table 1B:** Renal response at 1 year after the start of therapy, clinical and therapeutic characteristics at last observation in all patients, in those who developed lupus nephritis at <18 years, between >18 and <45 years, and >45 years of age.

	All patients	Missing data	LN diagnosis ≤18 years	LN diagnosis >18 and <45 years	LN diagnosis ≥45 years	P among the three age groups*	P between young and old class*
Renal response 1 year after the start of therapy							
Complete renal remission, n (%)	142 (54.6%)	14 (5.4%)	18 (39.1%)	100 (57.8%)	24 (58.5%)	0.07	0.07
Partial renal remission, n (%)	67 (25.8%)	14 (5.4%)	18 (34.8%)	42 (24.3%)	9 (22%)	0.10	0.08
No renal response, n (%)	37 (14.2%)	14 (5.4%)	6 (13%)	24 (13.9%)	7 (17.1%)	0.84	0.60
Therapy at the last observation							
Maintenance prednisone dosage (mg/day)	5 (0; 5)	0	1.88 (0; 5)	5 (0; 5)	5 (0; 5)	0.28	0.68
Free from corticosteroids, n (%)	81 (31.2%)	0	18 (39.1%)	54(31.2%)	12 (29.3%)	0.54	0.33
IS maintenance, n (%)	150 (57.7%)	0	27 (58.7%)	101 (58.4%)	24 (58.5%)	1.00	0.99
Cyclophosphamide, n (%)	2 (0.8%)	0	0	2 (1.2%)	0	**/**	1.00
Azathioprine, n (%)	24 (9.2%)	0	5 (10.9%)	15 (8.7%)	4 (9.8%)	0.89	0.86
Mycophenolate, n (%)	110 (42.3%)	0	20 (43.5%)	72 (41.6%)	18 (43.9%)	0.95	0.97
Cyclosporine, n (%)	11 (4.2%)	0	1 (2.2%)	9 (5.2%)	2 (4.9%)	0.68	0.49
Methotrexate, n (%)	3 (1.2%)	0	0	3 (1.7%)	0	**/**	1.00
Hydroxychloroquine, n (%)	168 (64.6%)	0	29 (63%)	113 (65.3%)	26 (63.4%)	0.95	0.97
Belimumab, n (%)	30 (11.5%)	0	4 (8.7%)	26 (15.0%)	0	**/**	1.00
Data at last observation							
Months of FUP	177.5 (84.75; 295)		254 (120; 385.5)	178 (84; 291)	135 (56; 232)	<0.01*	<0.01*
Serum creatinine (mg/dl)	0.84 (0.7;1.1)	0	0.89 (0.77; 1.13)	0.82 (0.69;1.04)	0.9 (0.78; 1.2)	0.87	0.98
eGFR (ml/min/1.73 m^2^; according to CKD-EPI)	90.59 (66.42, 107.29)	0	96.71 (64.39, 112.61)	94.57 (71.14, 109.09)	74.4 (48.12, 86.78)	<0.01*	0.01*
Proteinuria, g/day	0.19 (0.10, 0.55)	0	0.20 (0.10, 0.52)	0.20 (0.10, 0.57)	0.20 (0.09, 0.41)	0.95	0.29
Proteinuria >3.5 g/day, n (%)	6 (2.3%)	0	1 (2.2%)	5 (2.9%)	0	/	1.00
CKD, n (%)	54 (20.8%)	0	11 (23.9%)	31 (17.9%)	12 (29.3%)	0.23	0.57
SDI	1 (0,2)	13 (5%)	1 (0,1)	1 (0,2)	2 (1,3)	0.99	0.02*
Delta SDI/year	0 (0, 0.072)	13 (5%)	0 (0, 0.030)	0 (0, 0.066)	0.1 (0, 0.164)	1	0.12
Death, n (%)	14 (5.4%)	0	0	7 (4%)	7 (17.1%)	/	1.00

n°, number; pts, patients; FUP, follow-up; eGFR, estimated glomerular filtration rate; CKD, chronic kidney dysfunction; SDI, SLICC Damage Index; SLICC, systemic lupus international collaborating clinics American College of Rheumatology Damage index. If not differently specified data are expressed as median (first and third quartiles). Delta SDI/year: has been calculated by dividing the SLICC increase (the difference between the value at the last observation and the baseline) by the number of years of observation.

*P refers to T-test for continuous measurements between the first and third age class, Kruskal–Wallis H test for continuous measurements among the three classes, and to Chi-squared test for discrete measurements, with P<0.05.

At the last observation, after a median follow-up of 14.8 years (7-24.6), 54 patients (20.8%) had CKD (of them, 19 [7.3%] needed renal replacement therapy), and 14 patients (5.4%) died. Of the 14 deaths, only one was directly related to SLE and due to pulmonary hypertension. 31% of patients were free from corticosteroids, 51.7% were on maintenance immunosuppression, 11.5% were on belimumab therapy, and 64.6% were on hydroxychloroquine (**Table 1B**). There were 85 patients (32.7%) who had no chronic damage (SDI=0), and the median SDI was 1 (0-2).

### 3.2 Comparison between the three age groups

At LN diagnosis, 46 (17.7%) patients were <18 years old, 173 (66.5%) between 18 and <45 years, and 41 (15.8%) ≥45 years. There were no differences in gender, amount of proteinuria, hematuria, histological classes at kidney biopsy, and SLEDAI (**Table 1A**). The time between SLE and LN diagnosis was shorter in children (0 [0–4.75] months) than in adults (9 [0–71] months) and old patients (19 [0–132] months P= 0.001). Acute kidney disease (AKD) was significantly more prevalent in elderly patients (46%) compared with children (32.6%) and adults (24.3%; P = 0.02). Children had lower C3 (55 [34.75–80] mg/dl) and C4 (8 [4-13.5] mg/dl) and more frequent fever at LN diagnosis (69.6% of patients) in comparison with both adults (C3:56 [46-73]; C4: 10 [6-14] mg/dl, fever: 49.7%) and older patients (C3: 68 [50-83]; C4: 10 [5-18] mg/dl, fever: 29.3% P=0.001). In the elderly, the chronicity index at kidney biopsy was significantly higher (3 [1-4]) than in pediatrics (0 [0-2] P= 0.001) and in adults (1 [0-3] P= 0.001) (**Table 1A**), as well as the SDI (elderly 0 [0-1] vs. pediatrics 0 [0-0.25], P<0.001, and vs. adults (0 [0-1], P=0.026).

Again, in the elderly, arterial hypertension (78% vs. 46.2% of adults and 41.3% of children P=0.001) and anti-SSA antibody positivity (51.2% vs. 31.8% in adults and 15.2% in children P=0.001) were significantly more frequent. There were no significant differences in induction therapy among the three groups; iv MPP was given to 82.6% of children, 80.3% of adults, and 92.7% of older patients (P= 0.17). Among the immunosuppressive drugs, 47.8% of children, 44.5% of adults, and 41.4% of the elderly received cyclophosphamide (P=0.83). Mycophenolate mofetil was more frequently used in the elderly (31.7%), than in adults (19.7%) and children (13% P=0.42) (**Table 1A**).

After 1 year of induction therapy, the achievement of complete, partial remission, and no response were not significantly different among the three groups. At the last observation, eGFR was significantly lower in the elderly (74.4 [48.1-86.8] ml/min) than in adults (94.6 [71.1109.1]mil/min) and pediatric patients (96.7 [64.4-112.5] ml/min P= 0.001) (**Table 1B**).

A total of 11 children (23.9%), 31 adults (17.9%), and 12 older patients (29.3%) had CKD (P=0.56) at the end of follow-up. Only 19 patients with CKD (35.2%) had end-stage kidney disease (eGFR < 15 ml/min), and all started hemodialysis, without significant differences among age groups (P = 0.61). Four out of the 19 patients who started hemodialysis received a deceased kidney transplant (all the three children and one of the adult group). No deaths were observed in children, whereas seven adults (4%) and seven older patients (17.1%) died. The median SDI was significantly higher in the older group when compared with children (2 vs. 1; P=0.02) (**Table 1B**).

**Figure 1:**
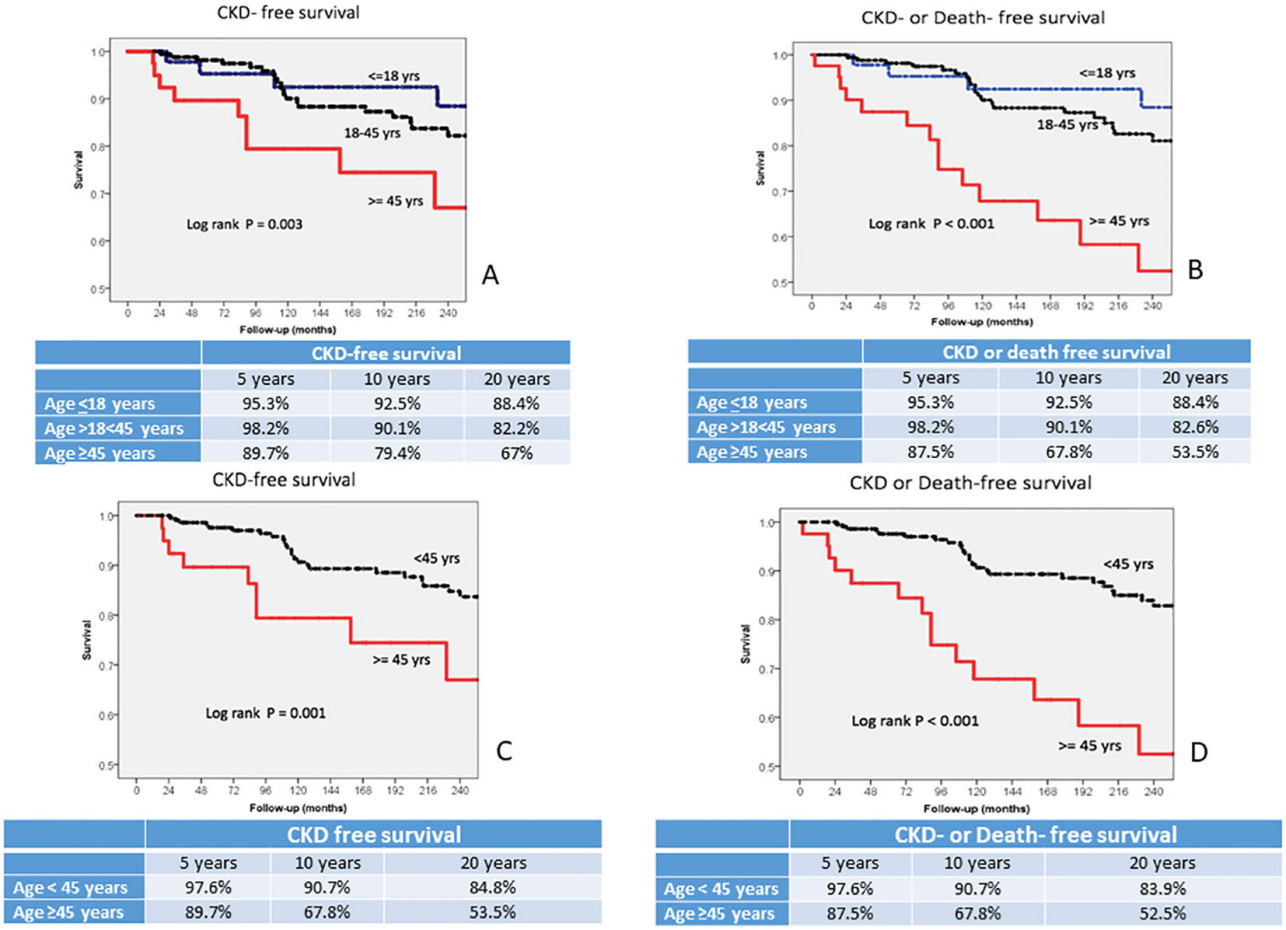
**(A, B)** CKD-free survival and CKD or death-free survival in lupus nephritis (LN) patients with different classes of age at LN diagnosis: age <18 years, age >18 and <45 years, age ≥45 years. **(C, D)** CKD-free survival and CKD or death-free survival in LN patients with <45 years of age at LN diagnosis and ≥45 years. CKD, chronic kidney disease.

Kaplan–Meier CKD-free survival curves demonstrated significant differences among the three groups. CKD-free survival at 5, 10, and 20 years were 95.3%, 92.5%, and 88.4% for children, 98.2%, 90.1%, and 82.2% in adults, and 89.7%, 79.4%, and 67% in the elderly (P=0.003, **Figure 1A**), respectively, and similar significant differences were obtained when CKD or death-free survival curves of three age groups were compared (P=0.001, **Figure 1B**). However, when analyzed separately, there was no significant difference in CKD-free survival between children and adults (P=NS), whereas both groups had significantly better survival than the elderly (P=0.002 for children, and P=0.004 for adults). To highlight the difference among age groups, a CKD-free survival curve including children and adults (group aged <45 years) was compared with that of the elderly. The survival at 5, 10, and 20 years of patients aged <45 years were 97.6%, 90.7%, and 84,8%, in comparison with 89.7%, 67.8%, and 55.5% of the elderly (P=0.001 **Figure 1C**), respectively. **Figure 1D**, which reported the differences in CKD or death-free survival between patients aged <45 years and those aged ≥45 years, makes more visible the differences between the two groups (P<0.001).

### 3.3 Predictors of CKD and CKD or death in the whole cohort

To define the importance of age groups in predicting the long-term LN outcome (CKD and CKD or death), we performed a Cox regression analysis to identify predictors of these two endpoints in our entire cohort of LN patients. The baseline variables entered in the COX regression analysis are reported in **Supplementary Table 1**.

As reported above, age classes and chronicity index at kidney biopsy were directly and significantly correlated. To better define their values on discriminating the outcomes, these two variables were considered one at a time in the Cox regression analysis.

#### 3.3.1 Predictors of chronic kidney disease

At multivariable analysis, AKD at diagnosis (relative risk, RR: 2.939; CI: 1.510-5.721; P=0.002), SDI >0 (RR: 1.824; CI: 1.155-2.881; P=0.010), arterial hypertension (RR: 2.752; CI: 1.316-5.754; P=0.007), and no remission 1 year after the start of treatment (RR: 4.316; CI: 2.008-9.277; P<0.001) were the independent variables associated with CKD development. Induction treatment with methylprednisolone pulses (MPPs) was associated with a risk reduction of CKD by 59% (RR: 0.409; CI: 0.206-0.811; P=0.011) (**Table 2A**). Although not significant (P=0.075), the chronicity index increased the risk of CKD by 10% for each additional unit calculated at kidney biopsy.

**Table 2A:** Multivariable Cox regression analysis (Backward method) performed on the whole cohort of patients to identify predictors of CKD.

Multivariable analysis						
	B	SE	Wald	RR	95% RR CI	P
AKD *	1.078	0.340	10.061	2.939	1.510-5.721	0.002
SDI > 0	0.601	0.233	6.639	1.824	1.155-2.881	0.01
Arterial hypertension	1.012	0.376	7.236	2.752	1.316-5.754	0.007
Methylprednisolone pulses	−0.894	0.350	6.543	0.409	0.206-0.811	0.011
No remission at 1 year	1.462	0.390	14.025	4.316	2.008-9.277	0.000

SDI, SLICC Damage Index; SLICC, systemic lupus international collaborating clinics American College of Rheumatology Damage index.

*AKD= eGFR <60 ml/min/1.73/m^2^ for <3 months, hematuria (urinary red blood cells >10/high-power field [HPF]), and/or erythrocyte casts, proteinuria ≥0.5 g/day.

B, beta coefficient; SE, standard error of beta coefficient; Wald, Wald statistic; RR, relative risk; RR 95% CI, 95% confidence intervals of RR.

#### 3.3.2 Predictors of chronic kidney disease or death

When both age and chronicity index were included in the model, both were not correlated with the endpoint of CKD or death (**Supplementary Table 2**). Therefore, we built two different models, the first including age (**Table 2B**) and the second including chronicity index (**Table 2C**). In the first model, older age in comparison with childhood age increased the probability of CKD or death by 3.278 (CI: 1.402–7.662: P=0.006). In the second model, the chronicity index predicts the endpoint with a relative risk of 1.136 for each additional unit (CI:1.021-1.264: P=0.019). In both models, the other variables independently associated with this endpoint (**Tables 2B, C**) were AKD, arterial hypertension, and no remission at one year.

**Table 2B:** Multivariable Cox regression analysis (Backward method) performed on the whole cohort of patients to identify predictors of CKD or death including in the model age classes but excluding chronicity index.

Multivariable analysis						
	B	SE	Wald	RR	95% RR CI	P
Age *	1.187	0.433	7.508	3.278	1.402-7.662	0.006
AKD **	1.075	0.286	14.155	2.930	1.674-5.130	0.000
SDI > 0	0.601	0.233	6.639	1.824	1.155-2.881	0.01
Arterial hypertension	1.306	0.354	13.607	3.692	1.844-7.389	0.000
No remission at 1 year	1.565	0.362	18.745	4.784	2.355-9.716	0.000

*Comparison between older patients and pediatrics.

**AKD = eGFR <60 ml/min/1.73/m^2^ for <3 months, hematuria (urinary red blood cells >10/high-power field [HPF]), and/or erythrocyte casts, proteinuria ≥0.5 g/day.

SDI, SLICC Damage Index; SLICC, systemic lupus international collaborating clinics American College of Rheumatology Damage index.

B, beta coefficient; SE, standard error of beta coefficient; Wald, Wald statistic; RR, relative risk; RR 95% CI, 95% confidence intervals of RR.

**Table 2C:** Multivariable Cox regression analysis (Backward method) performed on the whole cohort of patients to identify predictors of CKD or death including in the model chronicity index but excluding age classes.

Multivariable analysis						
	B	SE	Wald	RR	95% RR CI	P
AKD *	1.080	0.327	10.930	2.945	1.552-5.588	0.001
Chronicity index	0.128	0.054	5.519	1.136	1.021-1.264	0.019
Arterial hypertension	0.981	0.368	7.104	2.668	1.297-5.491	0.008
No remission at 1 year	1.555	0.382	16.535	4.735	2.238-10.020	0.000

*AKD= eGFR <60 ml/min/1.73/m^2^ for <3 months, hematuria (urinary red blood cells >10/high-power field [HPF]), and/or erythrocyte casts, proteinuria ≥0.5 g/day.

B, beta coefficient; SE, standard error of beta coefficient; Wald, Wald statistic; RR, relative risk; RR 95% CI, 95% confidence intervals of RR.

## 4 Discussion

In this large Italian cohort of LN patients followed for 14 years, we examined clinical presentation, patient, and kidney survival across three ages at LN diagnosis: childhood, adulthood, and advanced age. SLE mainly affects women during reproductive ages but can also occur in children and the elderly (1–5). We divided patients into these three groups: early-onset, adult-onset, and late-onset groups. Patients in the early onset group were younger than 18 years old at LN diagnosis. Our choice is consistent with that of other published papers. Mean age at diagnosis of our early-onset group was 14.8 + 3.6 years, very similar to the 13.7 + 3.3 years in Chan et al.’s paper (24), and the 14.2 + 2.39 years in Kang et al.’s study (25). Patients belonging to the adult-onset group were older than 18 at diagnosis, yet younger than 45. The late-onset group comprises patients older than 45, which was held as cutoff considering the average menopausal age of our cohort, in line with previous papers (26).

LN was diagnosed before the age of 18 in 17.7% of patients and after the age of 45 in 15.8%. Our results align with similar studies available in the literature, in which the authors compared LN patients categorized into different groups based on age at clinical LN diagnosis, with 15%-20% of cases occurring in pediatric patients and up to 10%-15% in elderly patients (25, 28, 29). The relatively small amount of patients receiving antimalarials at disease onset in our cohort may be due to the historical nature of the cohort covering a remarkable timespan, up to when the use of antimalarials was not usual in LN.

Children and the elderly had more frequent AKD at diagnosis than adults. In children, AKD was linked to low complement and extrarenal SLE manifestations, in the elderly, to arterial hypertension, high chronicity index at kidney biopsy, and SDI. Furthermore, most children had simultaneous SLE and LN diagnoses, whereas in 40% of the elderly, LN was diagnosed after >5 years of SLE. Therapy did not differ across groups. The 20-year kidney survival rate in children (88.4%) was comparable with adults (82.2%; P = NS) but significantly better than in elderly patients (67%; P = 0.002 for children vs. elderly, P = 0.004 for adults vs. elderly). These findings underscore the potential for early, aggressive management of lupus nephritis in younger patients to preserve long-term kidney function, while highlighting the challenges of managing advanced chronic damage in elderly patients. The same differences were obtained when CKD or death was the endpoint considered.

Most pediatric studies confirmed frequent simultaneous SLE and LN onset (11, 24), but different results are reported in the elderly. Xu et al. (10) confirmed our long SLE duration before LN, although another cohort (29) showed a short interval between SLE and LN. A long duration of lupus before nephritis causes chronic damage secondary to SLE activity, therapy, and age-related comorbidities (30, 31). This can explain why in some studies, in keeping with our data, the chronicity index and SDI were higher, and arterial hypertension more frequent in older patients (10, 25, 32). These features are well-known predictors of worse renal prognosis (33, 34). On the other hand, SLE children often showed active but potentially reversible disease with more frequent hypocomplementemia, higher SLEDAI, and hematologic and neuropsychiatric manifestations (6, 25, 35, 36).

In keeping with our results, Kang et al. (25) in a cohort of 117 Korean patients followed for 76.5 months reported CKD in 10% of children, 28% of adults, and 54.4% of the elderly. In 52 Turkish childhood-onset LN patients followed for 43.1 months, kidney survival at 10 years was 85.7%, a little worse than our 92.5% (37). In 92 LN Chinese children, the outcome that included CKD, ESKD, or death was 83.2% at 20 years (24). These good results confirm a significant improvement in the survival of LN children in the last decades even if the response to therapy at 1 year continues to be reported as suboptimal, several patients developed SLE flares, and the standardized mortality ratio continued to be higher than in adults (18.3 compared with a ratio of 3.1) (27, 35).

It is debatable whether late-onset LN is associated with a different disease course and prognosis than early-onset LN. Mongkolchaiarunya et al. (38) found no significant difference in response at 1 year in 30 late-onset vs. 90 early-onset LN, although mortality was higher in late-onset. In comparing 30 Chinese old-onset and 242 early-onset LN patients, the early-onset group seemed to have a poorer renal outcome after 6 years of observation (10). Accordingly, in a large retrospective cohort study from the NHIR Taiwan Database, the risk of incident ESRD was statistically lower in late-onset SLE patients compared with adult-onset patients (39). A large Chinese LN cohort (28), which included 102 late-onset and 1,162 early-onset patients followed for 55 months, confirmed the significantly higher mortality at 10 years in late-onset, but the kidney survival was not significantly different between the two groups. In our cohort, at the last observation, the percentage of CKD was higher in the elderly than in adults and children, but the differences were not significant. Kaplan–Meier survival curves showed no significant differences in CKD and CKD or death-free survival at 20 years between children and adults whereas both age groups had significantly better survival than the elderly. These differences became more evident at a survival curve summarizing the data of children and adults compared with those of the elderly. Of note, this is the longest follow-up reported in the literature, and it is not unexpected that with such a longer observation, the baseline chronic damage, arterial hypertension, and the natural loss of renal function with age together with age-related morbidity caused an increased rate of CKD. Moreover, our results confirmed the above-reported higher mortality in the elderly than in children and in adults (28, 38). The higher mortality in the elderly group may also be explained by the fact that elderly patients are more vulnerable to immunosuppressives compared with younger adults and children.

To emphasize the importance of age in kidney outcomes, we performed a multivariable analysis to identify predictors of CKD and CKD or death in the entire population. In the Cox model, chronicity index and age were tested separately because of a significant direct correlation between these two parameters. Regarding CKD predictors, we found that induction treatment with MPP was associated with a risk reduction of CKD by 59%. The importance of initial therapy with MMP pulses in active LN has been known since the seventies and was recently confirmed by EULAR and KDIGO recommendations (40–42). The other predictors of CKD were AKD, arterial hypertension, SDI >0 at baseline, and no complete response at 1 year. The association between SDI and mortality is reported in many studies (43, 44), and our data underline the important role of baseline chronic damage in predicting CKD. Age was not associated with CKD probably for the low number of events. Indeed, when we considered CKD or death as the endpoint, age at old-onset LN compared with child-onset was an independent predictor. AKD, arterial hypertension, and no response at 1 year were the other independent predictors in multivariable analysis. Impaired renal function and arterial hypertension at diagnosis are well-known predictors of bad long-term renal outcomes (18, 45, 46) as well as the lack of response to therapy (47–49). Limitations include retrospective design, a smaller sample of children and elderly, non-randomized therapy, long period of nephritis diagnosis during which the therapeutic approach is modified. The lack of data about SLE treatment in patients with a disease duration >1 year before LN diagnosis may have precluded to establish the prognostic role of some drugs. Although the predominance of Caucasian patients in our cohort reduces the possibility of extending these results to other ethnicities, this may represent a benefit considering that both genetic and environmental factors play a crucial role in the development and progression of diseases. Consequently, research in this field underscores the importance of conducting nationwide and ethnic studies, considering differences rather than similarities in their results.

Despite the limitations, we have demonstrated comparable long-term kidney survival in children and adults, likely due to early diagnosis and aggressive treatment. Elderly LN patients had the worst outcomes, possibly due to chronic damage at diagnosis and prolonged SLE duration before LN. Considering the recent demonstration that LN tended to appear at a more advanced age (41) and, given the poorer prognosis in late-onset lupus nephritis patients, tailored diagnostic and therapeutic strategies are necessary to mitigate chronic damage and improve long-term outcomes.

## Data Availability

The raw data supporting the conclusions of this article will be made available by the authors, without undue reservation.
