# Supplementary material for: Long-term prognosis of lupus nephritis: comparison between pediatric, adult, and advanced age onset
Source: Front Immunol. 2025 Mar 13;16:1531675. doi: 10.3389/fimmu.2025.1531675 (PMC11966454; doi:10.3389/fimmu.2025.1531675)
Supplement: Supplementary file 1 [file Table1.docx]

**Supplementary Table 1:** Variables considered in Cox regression analysis (Backward method)

| Age | Evaluated as a continuous variable |
| --- | --- |
| Age 1 | Comparison between adult patients and pediatrics |
| Age 2 | Comparison between pediatrics and elderly |
| Methylprednisolone pulses | Methylprednisolone pulses as induction therapy |
| Proteinuria g/24h | Measured at the start of therapy |
| Delta of proteinuria at one-year g/day | The difference between proteinuria at baseline and after one year of therapy |
| eGFR ml/min per 1.73 m^2^ | with the CKD EPI formula for patients > 18 years old and using the modified Schwartz formula for pediatric patients |
| Acute kidney disease | eGFR <60ml/min/1.73/m^2^ for <3 months, haematuria (urinary red blood cells >5/high power field [HPF]), and/or erythrocyte casts, proteinuria ≥0.5g/day |
| SLEDAI at baseline | Evaluated with the Systemic Lupus Erythematous Disease Activity Index 2000 (SLEDAI-2K) |
| SLICC at baseline | Systemic lupus international collaborating clinics American College of Rheumatology Damage index |
| Chronicity index | Evaluated at basal kidney biopsy |
| Complete remission at one year after the start of therapy | eGFR>60 ml/min, proteinuria <0.5g/day, and inactive urinary sediment |
| Complete +partial remission at one yar after the start of therapy | Partial remission: eGFR >60 ml/min per 1.73 m^2^, and proteinuria <3.5g/day |
| Arterial hypertension at baseline | Systolic blood pressure >140 mm/Hg and/or diastolic blood pressure >90 mm/Hg in sitting position. |
|  |  |

**Supplementary Table 2**

Multivariable Cox regression analysis (Backward method) was performed on the whole cohort of patients to identify predictors of CKD or death including in the model both age and chronicity index.

| **Multivariable Analysis** | | | | | | |
| --- | --- | --- | --- | --- | --- | --- |
|  | **B** | **SE** | **Wald** | **OR** | **CI** | **P** |
| **Age*** |  |  | 5.075 |  |  | 0.099 |
| **Age**** | -.185 | 0.399 | 0.215 | 0.831 | 0.380-1.816 | 0.643 |
| **Age***** | 0.646 | 0.492 | 1.727 | 1.908 | 0.728-5.002 | 0.189 |
| **Methylprednisolone pulses** | -1.032 | 0.360 | 8.195 | 0.356 | 0.176-0.722 | 0.004 |
| **AKD****** | 1.098 | 0.337 | 10.608 | 2.998 | 1.548-5.805 | 0.001 |
| **Chronicity Index** | 0.104 | 0.057 | 3.284 | 1.109 | 0.992-1.241 | 0.070 |
| **Arterial hypertension** | 1.177 | 0.384 | 9.381 | 3.246 | 1.528-6.895 | 0.002 |
| **No remission at 1 year** | 1.564 | 0.387 | 16.368 | 4.779 | 2.240-10.197 | 0.000 |

Age* as a continuous variable

Age**: Comparison between adult and pediatric patient

Age***Comparison between older and pediatric patients

****AKD = acute kidney disease defined as = eGFR <60ml/min/1.73/m^2^ for <3 months, haematuria (urinary red blood cells >5/high power field [HPF]), and/or erythrocyte casts, proteinuria ≥0.5g/day
